# Estimating the need for inpatient neonatal services: an iterative approach employing evidence and expert consensus to guide local policy in Kenya

**DOI:** 10.1136/bmjgh-2017-000472

**Published:** 2017-11-14

**Authors:** Georgina A V Murphy, Donald Waters, Paul O Ouma, David Gathara, Sasha Shepperd, Robert W Snow, Mike English

**Affiliations:** 1 Centre for Tropical Medicine and Global Health, Nuffield Department of Medicine, University of Oxford, Oxford, UK; 2 KEMRI - Wellcome Trust Research Programme, Nairobi, Kenya; 3 Oxford University Clinical Academic Graduate School, Medical Sciences Division, University of Oxford, Oxford, UK; 4 Nuffield Department of Population Health, University of Oxford, Oxford, UK

**Keywords:** child health, health services research, health policy, public health

## Abstract

Universal access to quality newborn health services will be essential to meeting specific Sustainable Development Goals to reduce neonatal and overall child mortality. Data for decision making are crucial for planning services and monitoring progress in these endeavours. However, gaps in local population-level and facility-based data hinder estimation of health service requirements for effective planning in many low-income and middle-income settings.

We worked with local policy makers and experts in Nairobi City County, an area with a population of four million and the highest neonatal mortality rate amongst counties in Kenya, to address this gap, and developed a systematic approach to use available data to support policy and planning. We developed a framework to identify major neonatal conditions likely to require inpatient neonatal care and identified estimates of incidence through literature review and expert consultation, to give an overall estimate for the year 2017 of the need for inpatient neonatal care, taking account of potential comorbidities.

Our estimates suggest that almost 1 in 5 newborns (183/1000 live births) in Nairobi City County may need inpatient care, resulting in an estimated 24 161 newborns expected to require care in 2017. Our approach has been well received by local experts, who showed a willingness to work together and engage in the use of evidence in healthcare planning. The process highlighted the need for co-ordinated thinking on admission policy and referral care especially in a pluralistic provider environment helping build further appetite for data-informed decision making.

**Key questions**

**What is already known about this topic?**

- Global under-five deaths have halved in the past 20 years; however, reductions in neonatal mortality rates have lagged greatly behind other advances.
- Many global estimates exist for individual causes of neonatal morbidity and mortality, but do not provide information of sufficient granularity for understanding the number of cases expected to require hospital care.
- Without this information, local policy makers are unable to make informed decisions regarding health service planning and monitoring, limiting the potential for sufficient neonatal mortality reductions to reach the Sustainable Development Goals.

**What are the new findings?**

- To our knowledge, this is the first attempt to estimate the number of neonates requiring inpatient care for a specific African population that incorporates evidence, local expert views and extant thinking on local policy.
- Our estimates suggest that almost one in five newborns in Nairobi City County may need inpatient care.
- Local experts actively engage and provide important local policy and contextual insights to inform integrated thinking on health service planning, and demonstrate a desire to engage in the ongoing transition to data-informed decision making.

**Recommendations for policy**

- We highlight the need for considerable strengthening and integration of health information systems and accurate, timely and granular analyses that should enable effective service provision and tailoring it to needs of high-risk areas.
- Before this is achieved, innovative use of available data informed by local experts may guide health policy and planning, enabling an important movement towards greater evidence-informed policy and service planning policy in low-resource settings.

## Introduction

Despite major achievements in many areas, the majority of countries in Africa did not make sufficient progress to meet their fourth Millennium Development Goal targets to reduce child deaths by two thirds between 1990 and 2015. In part, this is due to a smaller reduction in neonatal mortality (deaths in the first 28 days of life), which now accounts for over 40% of all child mortality in many countries.^1^ To meet the new Sustainable Development Goals, with all countries aiming to reduce neonatal mortality to at most 12 per 1000 live births,^2^ significant emphasis on, and progress in, reducing neonatal mortality is required.^3^

Many of the causes of mortality and long-term morbidity, for example, preterm birth; intra-uterine growth restriction; intrapartum-related neonatal encephalopathy; and infection can be tackled through universal access to basic high-quality health services delivered as part of essential facility-based inpatient care.^4–7^ However, knowing where and how to strengthen health services is limited by a lack of data to inform planning.^8^

In Nairobi City County, Kenya, neonatal mortality is estimated to be almost double the national average (39 compared with 22 per 1000 live births, respectively),^9^ but discussion with policy makers indicated that little is known about the volume of care that might be needed to reduce such high mortality. Indeed, absence of data undermines the development of any co-ordinated policy on admission or referral criteria and any efforts to plan future service scale-up, location or configuration. To address this practical need, we worked with a local expert group, drawing on available published and unpublished incidence estimates, while taking account of local policies, expertise and practices, to provide a credible guide to the magnitude of need for inpatient neonatal care in this large, urban setting. In this report, we describe our experiences of these practical efforts to estimate potential demand for care, with future reports exploring supply of care.^10^

## Local data for healthcare planning

Ideally, data from comprehensive civil registration and vital statistics programmes, on health facility utilisation, and from cause of death notification should be combined to ascertain mortality and morbidity rates and access to services at a population level to support health policy planning.^11^ However, in many resource-limited settings, such information systems are weak and alternative approaches to harness data are required.^12^ In Kenya, attempts at collecting routine data from hospitals are ongoing through health management information systems, such as DHIS2.^12^ However, such routine data systems are limited by poor reporting, particularly from private-sector facilities, and absence of neonatal indicators.^12–14^ Additionally, they cannot capture information about neonates who do not access health facilities. Current systems need considerable strengthening and investment to allow for more accurate, timely and spatially granular data to improve service provision and tailor it to high-risk areas.

In the absence of good local data, one possible approach could be to use local or regional estimates from the many global efforts to describe disease and mortality among children, including neonates.^15 16^ Unfortunately, although these approaches have been valuable for national and international agenda setting, they do not provide information at the level of detail required to understand the number^17^ of cases expected to need hospital care, limiting their usefulness to local policy makers and healthcare planning. Current estimates also frequently define a condition by presence or absence rather than level of severity that would determine requirement for care. Comorbidities are also often poorly described in the literature, making it difficult to differentiate the number of newborns needing hospital care from the number of incident episodes.

## Expert-lead approach to estimating the need for neonatal care

In the absence of reliable local data or directly applicable estimates from the literature, we set out to work with a local expert advisory group to develop plausible and informative estimates of the requirement for inpatient neonatal care in Nairobi City County.

We established an advisory group of senior clinical epidemiologists, senior Ministry of Health and County personnel responsible for child health, and neonatologists from public and private sector hospitals in Nairobi City County (n=10, see Acknowledgements section). The group met for a full day of consultations on four occasions to discuss neonatal conditions that would require inpatient care, with facilitation provided by a senior researcher to develop consensus positions where needed.

### Neonatal admission policies in Kenya

**Figure 1:**
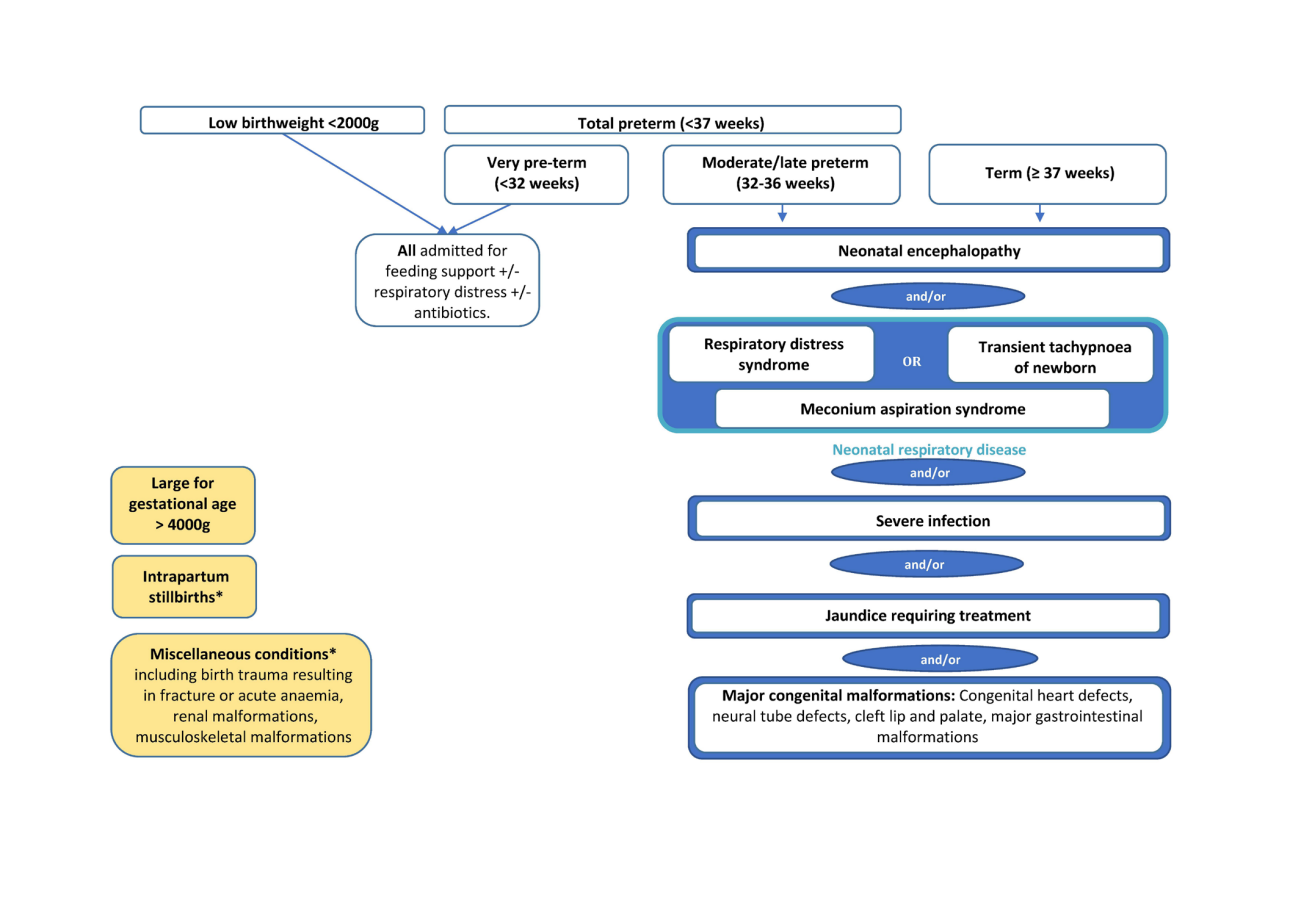
Admissions framework: conditions requiring neonatal inpatient care in Nairobi City County.

National guidelines on neonatal admission policies do not currently exist in Kenya, with different health facilities following varying practices (Murphy *et al*, unpublished). Hence, we worked with the advisory group to define the population that requires inpatient services. Many conditions were identified that could result in inpatient care requirements. However, in an effort to minimise the uncertainty of our overall estimates and to take a pragmatic approach, we focused only on major conditions (figure 1). The conditions identified were similar to conditions identified in most international settings, including for example severe infection and neonatal respiratory diseases. The advisory group also considered relevant local policies and practices, such as new Kenyan guidelines that advise that all newborns with a low birth weight (BW) <2000 g should be admitted for kangaroo mother care. Other examples of local practice decisions are outlined in table 1. Terminology of conditions in the framework (figure 1) are defined in table 2.

**Table 1:** Key discussions and decisions from local expert advisory group

Condition	Discussion points	Decision
**Birth weight <2000 g**	New guidelines are being produced by the Ministry of Health, which will indicate that all newborns <2000 g should be admitted for kangaroo mother care (KMC).	2000 g should be applied as the definition for low birth weight requiring admission as KMC and neonatal units are typically co-managed.
**Large for gestational age/foetal macrosomia**	All neonates >4000 g should be admitted for investigation of aetiology, and other support as required (such as feeding to prevent hypoglycaemia). However, this care is usually provided on the postnatal ward rather than the newborn unit.	Acknowledge in the framework but do not include in overall estimation.
**Neonatal encephalopathy**	Minor neonatal encephalopathy does not necessitate neonatal inpatient care and carries no long-term risk of neurological disability.	Only Sarnat grades II and III^42^ should be included in estimation.
**Neonatal respiratory diseases**	Neonatal respiratory distress syndrome (RDS) and transient tachypnoea of the newborn (TTN) are difficult to differentiate in many clinical settings in Kenya, and are often classified predominantly on gestational age. Management is largely consistent across these groups. Although meconium aspiration syndrome (MAS) is a distinct clinical entity, respiratory support management is similar to that of RDS and TTN.	A composite outcome of ‘neonatal respiratory diseases was created’, comprising all neonates with RDS, TTN and MAS requiring inpatient care. It was recognised, however, that ultimately distinguishing the different aetiologies of neonatal respiratory diseases will be important for health services.
**Late-onset neonatal sepsis**	Most neonates >7 days old with severe infection are likely to be admitted to the paediatric ward rather than the newborn unit, and so, from a health service provision perspective, should not be counted in our framework.	Attempts should be made to separately estimate early-onset (<7 days old) and late-onset neonatal sepsis.
**Jaundice**	A large number of jaundice cases will resolve without treatment. Jaundice requiring treatment is likely to be in the first week of life and be provided as an inpatient in the neonatal unit. On the other hand, jaundice in older neonates is likely to be investigated±treated as an outpatient or on the paediatric ward.	Only ‘jaundice requiring inpatient treatment’ in the first week of life should be included in the framework.
**Major congenital malformations**	Defining which congenital malformations require inpatient neonatal care is complex. Some malformations that might be diagnosed in the neonatal period in a high-resource setting may present later in the Nairobi population due to a combination of delayed diagnosis and care-seeking behaviours.	Only congenital malformations likely to result in mortality or severe morbidity without neonatal inpatient care (most commonly for surgical intervention) should be included in the framework. For details, see online supplementary appendix 1.
**Miscellaneous conditions**	There are other conditions that require inpatient care, potentially large in number but each with a low individual incidence and high level of uncertainty around their estimates, which should be acknowledged.	These conditions (specifically including birth trauma resulting in fracture or acute anaemia, and renal and musculoskeletal congenital malformations) should be acknowledged in the framework but not included in the overall estimation.

10.1136/bmjgh-2017-000472.supp1 Supplementary file 1

**Table 2:** Data sources for estimates of neonatal conditions requiring inpatient care

Condition	Definition/inclusion	Data source for crude estimate	Study population	Evidence level*	Data source for adjustment
**<32 weeks preterm**	All neonates born <32 weeks gestational age according to ‘best obstetric estimate’	Unpublished breakdown *(Zahida Quresh & Alfred Osoti, personal communication, 2016)* of published data from the WHO Multicountry Survey on Maternal and Newborn Health 43	All (n=6439) births in six hospitals in Nairobi City County May 2010–January 2011	V	NA
**Birth weight (BW)** **<2000** **g**	All neonates born with BW <2000 g				Birth data (n=5550) from Pumwani Maternity Hospital newborn unit *(Jalemba Aluvaala, personal communication, 2016)*
**Large for gestational age**	All neonates with BW >4000 g. The term *‘*large for gestational age*’,* rather than foetal macrosomia, is used in the framework as this is more commonly used within the Kenyan medical community				
**Neonatal encephalopathy**	Intrapartum-related hypoxia and its complications—Sarnat grades II and III^42^	Lee *et al*, 2013 44	Sub-Saharan Africa (SSA) modelled estimate for 2012 from global systematic review	IIIb	NA
**Neonatal respiratory diseases**	Respiratory distress syndrome (RDS), transient tachypnoea of the newborn (TTN) and meconium aspiration syndrome (MAS)	RDS/TTN 45 46 MAS* 47 48	RDS/TTN: Swedish population-based study of 481 416 neonates from 2004 to 2008 MAS: UK population-based study of 4 99 096 neonates from 1998 to 2000	IV	Gestational age breakdown^45 46^ ^47^
**Severe infection**	Possible severe bacterial infection (pSBI) as defined from WHO Young Infants Clinical Signs Study (YICCS) criteria)^31^	Seale *et al* 31	SSA modelled estimate for 2012 from global systematic review	IIIb	NA
**Jaundice requiring treatment**	Neonatal jaundice requiring medical intervention (ie, phototherapy or exchange blood transfusion)	Olusanya *et al* 49	5266 neonates presenting to four primary healthcare clinics for routine vaccination in Lagos, Nigeria. Overall vaccine uptake estimated to be 75%–98%. These clinics known to account for >75% of all vaccination in the city	V	Systematic review for the UK National Institute of Clinical Excellence^50^
**Major congenital malformations**	Congenital malformations likely to result in mortality or severe morbidity without neonatal inpatient care: congenital heart defects, major central nervous system defects, orofacial clefts, major gastrointestinal malformations	Different sources were used to estimate different malformation groups. Data were used from the Modell Global Database of Congenital Disorders^51 52^ the International Clearinghouse for Birth Defects Surveillance and Research, (ICBDSR)^53^ the European Concerted Action on Congenital Anomalies and Twins, (EUROCAT)^54^and individual studies from SSA^55–62^ and high-income settings.^63 64^ For details, see online (supplementary appendix table 2)		IIIa/IV/V	Prevalence ratios^64^ (large collection of USA birth registries: n=7, 209, 768 births) used to distribute cases by gestational age
**Intrapartum stillbirths**	Foetal death occurring during the period of labour, in neonates≥1000 g BW or ≥28 weeks of gestation	Lawn *et al* 65 and Cousens *et al* 66	Kenyan national stillbirths (total) estimate from global systematic review for 2009 and SSA estimate of proportion of stillbirths that are intrapartum for 2009^65 66^	IIIa/IIIb	NA

*Evidence levels correspond to the hierarchy of evidence outlined in appendix 2 table s1

†Balchin et al estimate incidence of meconium stained amniotic fluid (MSAF), Fanaroff reports only 5% of neonates born with MSAF develop MAS—results calculated accordingly (see online supplementary appendix 1 for details).

‡Although Seale et al focused on pSBI, the broad nature of the YICCS criteria they applied to identify pSBI also results in the inclusion of severe non-bacterial infections (i.e. viral or fungal), which may require inpatient neonatal care.

The advisory group also agreed to exclude some conditions (shown in yellow in figure 1 and described as ‘miscellaneous conditions’ in table 1), for example uncommon congenital malformations and birth trauma resulting in fracture or acute anaemia, felt likely to yield imprecise estimates of incidence. Intrapartum stillbirths were estimated separately with the understanding that, as intrapartum care improves, some of these deaths may be avoided and, resultantly, a proportion of these surviving newborns may require inpatient newborn services.

### Literature review of neonatal morbidity

The advisory group also discussed and approved a classification of evidence: an order of relevance by which available incidence estimates for neonatal conditions should be considered for the Nairobi setting (see onlineappendix 2 table s1). The ideal (level 1) was agreed to be population-based estimates for Nairobi City County. In the absence of such evidence, categories were, in order of relevance, as follows: level 2, large population-based estimates from populations similar to Nairobi City County; level 3, systematic reviews of population-based studies providing national or regional estimates; level 4, individual population-based studies of populations substantially different to Nairobi City County; and level 5, facility-based studies.

We conducted an iterative literature search up to 1 September 2015 using Medline, Embase, Global Health, Google Scholar and the WHO databases for incidence estimates for each of the selected conditions.^17^ We supplemented the search by contacting experts (see Acknowledgements section) for advice on published and unpublished literature, and by reviewing morbidity/mortality estimation papers from the Global Burden of Disease project and the accompanying bibliographies.^18 19^ Our aim was to identify the most relevant literature, guided by our classification and (see online supplementary appendix 2 table s1) advisory group.

After classifying, we assessed our certainty in the derived estimates using the structured parameters of GRADE (‘directness’, ‘risk of bias’, ‘imprecision’ and ‘inconsistency’).^20^ To do this, we presented estimates derived from the literature to the advisory group for discussion, agreement on the certainty of the condition estimates (as high, moderate, low or very low confidence in the appropriateness of the incidence estimate for the Nairobi population) and selection of estimates to use in our final analyses.

We followed the same process of literature review and expert group consultation to identify the likely overlap in estimates due to comorbidities. Where possible we adjusted estimates to ensure that newborns with multiple conditions were only counted once. For example, if a newborn had severe infection and was premature, we counted the newborn as needing one admission (either in the severe infection or preterm group). The specific approach to adjustment varied depending on the condition(s) being considered, these details are provided in the online supplementary appendix 1.

### Calculating need for inpatient services

We calculated the number of newborns likely to require care in 2017 by applying the estimate of the proportion of live births requiring inpatient neonatal services to the estimated number of live births in Nairobi City County in 2017. The number of live births was estimated by applying the Nairobi City County crude birth rate (3.1%) obtained from the Kenyan 2014 demographic and health survey^21^ to population estimates for the County, derived from the 2009 national census and adjusted for population growth from 2009 to 2017 at a rate of 3.89% per year.^22^ Population data by location were used to allocate births, and thus need for inpatient neonatal services, to areas within Nairobi City County and presented on a map. Locations are the lowest spatial scale for which variations in social inequalities are defined by the national government.^23 24^

## Number of newborns requiring inpatient care in Nairobi City County

We calculate that almost one in every five live born babies (183/1000 live births (lower and higher bounds of confidence: 148–221/1000 live births)) will require inpatient treatment in a newborn unit, where specialist care is predominantly focused on small and sick newborns in the first week of life. This number of newborns requiring inpatient care could vary from 149/1000 live births (126–176/1000 live births) to 207/1000 live births (169–249/1000 live births) depending on the specific admission criteria applied and the use of plausible lower and higher illness incidence estimates, respectively (see online supplementary appendix 2 table s3).

The estimated neonatal mortality for Nairobi City County is 39 per 1000 live births^21^; thus, our findings suggest that of those newborns requiring neonatal care (183 per 1000 live births), 21% currently die within the neonatal period.

The estimate of the total incidence of neonatal illness episodes for all selected conditions, without adjustment for comorbidity and double counting, was 223/1000 live births (183–266/1000 live births) (table 3). Hence, attempts to adjust for comorbidities reduced the estimated need for admissions by 18%. The leading causes of illness episodes were jaundice requiring inpatient treatment (predominantly in the first week of life) (73.9 (95% CI 67.1 to 81.2) per 1000 live births) and severe infection (62.0 (95% CI 41.0 to 83.0) per 1000 live births), accounting for 61% of the unadjusted estimate of neonatal conditions. Many of these newborns are likely to also have been preterm and/or BW <2000 g. Although not included in the overall calculation of newborns requiring admission, it was estimated that 10.2/1000 of births were intrapartum stillbirths.

**Table 3:** Unadjusted and adjusted incidence estimates of neonatal conditions requiring inpatient care

Conditions	Unadjusted estimate (bounds of confidence*) per 1000 live births	Adjusted estimates (bounds of confidence) per 1000 live births	Already excluded in unadjusted estimates†	Excluded by adjustment‡	GRADE§
<32 weeks preterm	14.60 (11.90–17.80)	14.60 (11.90–17.80)	TOP, GA <22 weeks, stillbirths	Not possible to adjust	**⊕⊕⊕◯**
BW <2000 g	31.22 (27.20–35.80)	14.31 (12.47–16.41)	Stillbirths	Those with other diagnoses in the framework	**⊕◯◯◯**
Neonatal encephalopathy	8.63 (6.10–13.30)	8.63 (6.10–13.30)	‘Where possible’ GA <34 weeks, BW <2000 g, severe infection, congenital malformations, stillbirths	Not possible to adjust	**⊕⊕⊕◯**
Neonatal respiratory diseases	24.56 (24.19–24.93)	17.09 (16.74–17.43)	RDS/TTN–GA <30 weeks, multiple pregnancies; MAS–GA <24 weeks, BW <500 g, multiple pregnancies, stillbirths	GA <32 weeks	**⊕⊕◯◯**
Severe infection	62.00 (41.00–83.00)	62.00 (41.00–83.00)	BW <500 g, GA <32 weeks, stillbirths	Not possible to adjust	**⊕⊕◯◯**
Jaundice requiring treatment	73.87 (67.10–81.20)	63.60 (57.70–69.91)	Stillbirths	Severe infection	**⊕◯◯◯**
Major congenital malformations¶	8.32 (6.07–10.02)	2.86 (2.31–3.42)	Nil	GA <32 weeks	**⊕⊕◯◯**
**Total**	223.19 (183.36–266.25)**	183.09 (148.10–221.46)**			

*95% CIs apart from in the case of ‘major congenital malformations’ (see supplementary appendix 1 for details).

†Excluded in primary data source.

‡Excluded following adjustment for overlap.

§Evaluation of confidence in estimates based on the GRADE framework.

¶Further details of the incidence estimates for major congenital malformation groupings are provided in online supplementary appendix 2 table S2.

**Lower and higher estimates calculated by summing lower and upper bounds of confidence, respectively.

**Figure 2:**
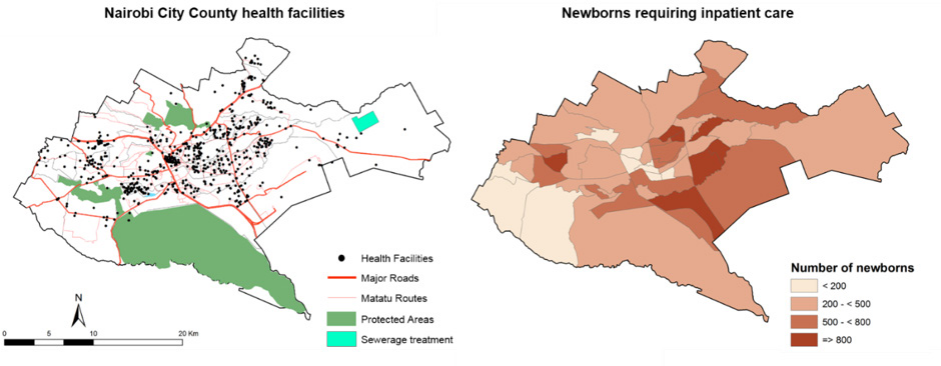
Distribution of newborns requiring inpatient care in Nairobi City County.

In the year 2017, the population of Nairobi City County is estimated as 4.26 million, with 132 025 live births occurring. Applying the estimate of 183/1000 live births, the total number of newborns in Nairobi City County requiring inpatient services is estimated to be 24 161 in the year 2017. Figure 2 shows the distribution of these newborn cases across the county by the administration unit of ‘location’. Under differing scenarios of admission (see online supplementary appendix 2 table s3), this number could range from 19 672 (149/1000 live births) to 27 329 (207/1000 live births).

### Local estimates in a global context

The estimate of 18.3% of newborns requiring inpatient care was considered credible by the expert advisory group, but uncertainty around the estimate was acknowledged. The estimate for Nairobi City County is higher than those found in high-income countries.^25–29^ In their 2016 report, the UK National Neonatal Audit Programme estimated that nearly one in eight babies (approximately 12.7%) born in England, Scotland and Wales were admitted to a Neonatal Unit.^28^ Hospitals in the USA participating in the National Perinatal Information Center/Quality Analytic Services admitted 14.4% of newborns to a special care nursery (neonatal immediate care units and neonatal intensive care units) from 1 July 2009 to 30 June 2010.^27^ The higher number of newborns requiring care in the Kenyan setting is plausible given more limited antenatal care, and higher incidences of low BW and preterm births and of often preventable conditions, such as severe infection.^4 15 30 31^ Indeed a large part of our estimated need for care is due to infection and jaundice (together accounting for 61% of illness episodes), indicating that better preventive care, infection control and improved care for jaundice in community facilities or even at home could considerably reduce the need for inpatient services in the long run.

## Challenges

### Weak evidence on neonatal morbidity

No population-based estimates for Nairobi City County or similar populations were identified in the literature. Most of the relevant sources of evidence were either from systematic reviews of population-based studies, providing regional estimates for East Africa or sub-Saharan Africa (neonatal encephalopathy and severe infection) or facility-based studies (prematurity, BW <2000 g and jaundice requiring treatment). We obtained estimates of the incidence of neonatal respiratory conditions from individual population-based studies in European populations. Several sources of evidence were used to estimate the incidence of major congenital malformations; these included systematic reviews of population-based studies providing modelled estimates for Kenya, population-based studies from high-income settings and facility-based studies.

Due to the lack of population-based estimates for Nairobi or similar populations, the certainty of all estimates was downgraded using the GRADE approach (see online supplementary appendix 2). Confidence in the estimates obtained from literature was further undermined by risk of bias (in particular facility-based studies that reported estimates for very preterm, BW <2000 g, jaundice and congenital malformations), imprecision (modelled estimates based on systematic reviews providing only regional level estimates with wide CIs) and inconsistency of estimates between studies (very preterm, BW <2000 g, jaundice and congenital malformations). Online supplementary appendix 3 provides further information on estimate specific limitations.

### Accounting for comorbidities

Table 3 outlines the exclusion of overlapping comorbid conditions through adjustment. For many conditions, complete adjustment for overlap between estimates due to comorbidity was not possible due to limited availability of estimates of comorbidity in the literature. This limitation may have led to an overestimate of the need for admission, with some newborns being counted more than once, particularly among those comorbid for prematurity and BW<2000 g. It is essential that future data systems accurately capture comorbidity to aid with accurate understanding of care requirements.

### Weak population data

Nairobi is a vast area encompassing the full range of at risk women, from the poorest in Kenya to the most affluent, resulting in marked heterogeneity in risk factors for neonatal outcomes.^32–35^ These communities of women are concentrated in specific areas of the County, with 60%–70% of the population estimated to live in slums.^35 36^ Population data on the demographic, socioeconomic and risk factor characteristics of the Nairobi population are, however, limited in their accuracy and granularity. We have used available population data at the ‘location’ level based on 2009 census data to calculate the number of live births in the County. However, there are variations in population density, social and economic factors, and access to services across the County that are likely to affect the distribution of at-risk populations and the incidence of poor neonatal outcomes.^32 33^ Planning services to meet discrete population needs requires much higher resolution of subsets of women at different levels of risk and need within the county boundaries.^37 38^

## Strengths

We have taken an innovative and pragmatic approach, encompassing local policy and practice, to providing information on burden of neonatal disease that can inform healthcare planning. Although limitations exist in the availability and quality of the data summarised for this purpose, before this analysis, the Nairobi City County government had no estimate of how much care may be required for their population, making our estimates a useful interim approach.

As far as we are aware, this is the first attempt to estimate the number of newborns requiring admission for a specific administrative population that also incorporates local expert views and extant thinking on local policy. Our approach is specifically aimed at providing information that can be used in support of future planning of health services to ensure universal access to basic quality care for small and sick newborns, and demonstrates applying published estimates (often indirect evidence) to a low-income setting. To support this effort, these results will complement ongoing work to evaluate the supply of neonatal services across public, private and not-for-profit sectors.^10^

As part of this process, we engaged with policy makers and healthcare planners at a local level in understanding the available information and its limitations, while also being sensitive to local policy. This engagement was valuable in its own right for demonstrating the need for careful thinking on policy (when and where to admit newborns), and recognition of the need for integrated thinking on health service planning, including referral pathways, that might span public, private and not-for-profit providers. Despite being at the forefront of healthcare planning and delivery, our experts had not come together previously to discuss these issues. It was clear that differences in practice across health facilities exist and there is a lack of clarity on policy for how neonatal care should be organised. As we have seen from our estimates (see online appendix 2 table s1) the number of newborns who should be admitted for care can vary widely depending on specific policies. Yet, the potential consequences of policy for the amount and type of care that needs to be delivered and the impact this might have on the overall organisation of services are typically ignored during the policy-making process.

## Conclusion

Universal access to quality healthcare and addressing high levels of neonatal mortality will be essential to meeting specific Sustainable Development Goals to reduce child mortality. Data for decision making are crucial for planning services and to monitor progress in these endeavours. Ideally, local population-level and facility-based data collection will, in the future, support accurate estimation of health service requirements. Initiatives to improve reporting rates and data quality, strengthen electronic data capture, and ensure data on neonates (and other neglected populations) are included in national systems should, therefore, be prioritised.^12 39–41^ There is an additional need to carefully consider how civil registration, vital statistics and health management information systems can be integrated, with linked investment in capacity to analyse and use these data. Before we achieve this, effective use of available data may guide policy and planning. Our approach has been well received by local experts, who showed a willingness to work together and a recognition of the importance of the use of evidence in healthcare planning in a resource-limited setting. These factors will prove important in the ongoing transition to data-informed decision making.
