# Supplementary material for: Estimating the need for inpatient neonatal services: an iterative approach employing evidence and expert consensus to guide local policy in Kenya
Source: BMJ Glob Health. 2017 Nov 14;2(4):e000472. doi: 10.1136/bmjgh-2017-000472 (PMC5687539; doi:10.1136/bmjgh-2017-000472)
Supplement: Supplementary file 1 [file bmjgh-2017-000472supp001.pdf]

Appendix 1: Supplemental methods

Overlap/co-morbidity adjustment

As this study's disease burden estimates are intended for policy development and planning of neonatal care provision, it was important to focus on *numbers of births requiring admission to the newborn unit*, in addition to the total number of cases of each disease. Therefore, as multiple diseases may co-exist during one admission, it was key to consider the overlap between different diseases and aim to avoid *multiple-counting* of admissions in neonates with comorbidity (e.g. very preterm and congenital malformations) where possible. For each condition, in addition to the overall estimate, a second estimate was developed with overlap excluded if possible (e.g. for the above example of congenital malformations we developed an estimate which excluded neonates who were also very preterm). It was only desirable to exclude overlap from one of any two categories, so as to avoid excluding neonates with comorbidity entirely. For the above example, a neonate born <32 weeks gestational age (GA) and also suffering from a major congenital malformation was excluded from the 'major congenital malformations' category but was counted in the 'very preterm' category. The process involved in adjusting estimates to exclude overlap varied depending on the condition, and is described below in the 'condition estimate calculations' section. It should be noted that our methodology aimed only to avoid double counting of individual neonatal admissions with more than one diagnosis, and not to exclude neonates with multiple separate admissions for different diagnoses (e.g. being admitted initially for prematurity, discharged, and then re-admitted with neonatal sepsis).

Condition estimate calculations

1. Very pre-term (<32 weeks)

Evidence source

Unpublished breakdown of published data from the World Health Organization Multicountry Survey on Maternal and Newborn Health (WHOMCS).[1] We utilised data from 6439 births in 6 hospitals in Nairobi City County (all births in these facilities between May 2010 and January 2011).[2]

The WHOMCS estimate was compared with two other potential sources of estimates. Based on their global systematic literature review, the Child Health Epidemiology Reference Group (CHERG) reported a modelled estimate of very pre-term births of 19.3/1000 live births for Kenya.[3] Another study of similar methodology by Marchant et al, which used more stringent, East-African evidence sources, modelled an estimate of 7.6/1000 live births (<34 weeks GA rather than <32 as in our framework) for East Africa.[4] It was decided during appraisal of these sources that the CHERG estimate should be interpreted with caution due to over half of the sources used for this meta-analysis not reporting the method of GA assessment used.[3] The study by Marchant et al may be an underestimate due to their exclusion of a group of neonates with missing data who also had a much higher neonatal mortality rate than that of the group of neonates included in the study.[4] The WHOMCS estimate lies centrally within the bounds of these two studies. Despite limitations due to inconsistency in GA estimation techniques and variance in estimates between hospital sites, WHOMCS was selected to be the most appropriate estimate for our purpose.

Calculations

Individual numbers of births <32 weeks GA reported for each of the 6 WHOMCS Nairobi hospitals [2] were summed and divided by the summed total number of births for each facility to give an overall incidence estimate. 95% confidence intervals were then calculated. In our framework, all neonates <32 weeks GA are assumed to be admitted regardless of presence of disease.

Overlap adjustment

The WHOMCS study excluded abortions, GA< 22 weeks, birthweight (BW) <500 g, and all stillbirths. It was not possible, with the available data, to adjust for overlap with any of the other conditions in the framework; however, in the majority of cases this was achieved through adjusting estimates for the other conditions to exclude very preterm neonates.

2. Birthweight <2000 g

Evidence source

Unpublished breakdown of published WHOMCS BW data [1] on 6439 births in 6 hospitals in Nairobi City County, as for 'very preterm' (see above).[2] These data allowed us to estimate low BW as defined as <2000 g. By contrast, all other studies on low BW identified only provided estimates for <2500 g.

Calculations

Individual numbers of births <2000 g BW reported for each of the 6 WHOMCS Nairobi hospitals were summed and divided by the summed total number of births for each facility to give an overall incidence estimate. 95% confidence intervals were then calculated.

Overlap adjustment

A large proportion of neonates who are <2000 g BW also have another admission diagnosis counted in the framework, and so it was attempted to exclude them from this estimate, as they are counted elsewhere. The WHOMCS data is not sufficiently detailed to provide an estimate of the proportion <2000 g with another admission diagnosis, and no other published evidence was identified which could help to derive this estimate. Resultantly, it was opted to adjust WHOMCS figures using unpublished data from neonates admitted to the NBU of Pumwani Maternity Hospital in Nairobi (unpublished, Aluvaala et al), the hospital with the most births each year in Nairobi (and all of Kenya). The ratio of births <2000 g BW admitted with no other diagnosis apart from diagnosis recorded as "prematurity or low BW" to overall births <2000 g BW admitted to the NBU was calculated as ratio= 0.458. This was then applied to the overall WHOMCS estimate of births <2000 g BW to calculate a new outcome excluding neonates <2000 g BW with another diagnosis at admission.

Unfortunately, using this method, it was not possible to exclude neonates <32 weeks GA reliably. Due to limitations in the ability to accurately assess GA, the Pumwani clinical admission proforma used has a combined admission diagnosis of "prematurity or low BW" rather than two separate categories. This will likely result in our adjusted outcome being an overestimate.

3. Neonatal encephalopathy

Evidence source

Sub-Saharan Africa (SSA) modelled estimate for 2012 from global systematic review by Lee et al,[5] adjusted as described below. This was selected as the most contextually appropriate estimate given the lack of population-level estimates for Nairobi or similar populations.

Calculations

To give an estimate of only Sarnat grade II and III neonatal encephalopathy, the overall neonatal encephalopathy incidence estimate for SSA of 14.9 per 1000 live births was multiplied by the sum of the proportion of grade II and III cases in countries with a neonatal mortality rate ≥15 (33.9% for grade II + 24.0% for grade III = 57.9%), also using data from Lee at al. This resulted in a revised estimate of 8.63 per 1,000 live births for neonatal encephalopathy grades II and III. The same multiplication factor was also applied to stated 95% confidence intervals to calculate these for the adjusted outcome.

Overlap adjustment

Of the seven SSA studies included in the Lee et al modelling, all specified that they exclude preterm birth, four excluded congenital malformations, and two excluded severe infection. As the review's cut-off for preterm birth complications was GA <34 weeks rather than our study's <32 weeks there is likely to be a proportion of neonates 32-33 weeks GA with neonatal encephalopathy who are missed in our estimates. Additional overlap may remain in our estimate due to some of the reviewed studies not excluding congenital malformations and no mention of efforts to exclude severe infection, neonatal jaundice or BW <2000 g across studies. Without detailed insight into the modelling techniques used, it was not possible to quantify this overlap or adjust the estimate. However, overlap between neonatal encephalopathy and BW <2000 g should have already been addressed through methods used to estimate BW <2000 g. Neonatal encephalopathy and neonatal respiratory distress syndrome (RDS)/ transient tachypnoea of the newborn (TTN) are thought of as distinct clinical entities with no overlap to consider between these conditions.[5] This is not the case for neonatal encephalopathy and meconium aspiration syndrome (MAS), for which overlap exclusion was not possible.

4. Neonatal respiratory diseases

Evidence source

Neonatal RDS and TTN estimates were taken from a population-based study of 481,416 neonates conducted in Sweden by Altman et al from 2004-2008.[6,7] MAS estimate was derived from a population-based study of 499,096 neonates conducted in the UK by Balchin et al from 1998-2000.[8] It was necessary to use high-income population-level studies as there were no robust estimates of these conditions for low-income settings. These high-income estimates were selected as the largest and most methodologically robust studies reporting incidence at a sufficient granularity.

Calculations

Balchin et al reported incidence of meconium stained amniotic fluid (MSAF), the vast majority of cases of which do not result in MAS. It was, therefore, decided to apply the frequently cited figure that 5% of neonates born with MSAF develop MAS [9,10] to these data to calculate the incidence of MAS. This displayed a biologically plausible J-shaped curve.[9,10] Incidence of RDS, TTN and MAS all vary markedly by GA. Resultantly, to calculate the overall outcome of 'neonatal respiratory diseases' for all GA, GA-specific incidences for each condition were identified. These GA-specific incidences

were applied to GA data from the WHOMCS study (see above) and summed to estimate overall incidence of neonatal respiratory diseases for all live births in Nairobi. Overall confidence intervals were calculated by summing the squared standard error for each estimate, taking the square root of this, and using normal approximation to build confidence intervals.[11]

Overlap adjustment

For RDS and TTN, estimates were available by week of GA from 30 weeks onwards and from 24 weeks onwards for MAS. All GA were included in the unadjusted outcome, however, neonates born <32 weeks GA were excluded when adjusting for overlap. The estimate for RDS/TTN from Altman et al already excluded multiple pregnancies. They did not report excluding neonates with severe infection, jaundice, congenital malformations, or BW <2000 g [6,7]. Balchin et al used for MAS reports excluding multiple pregnancies and births <500g. There is no mention of excluding neonates with severe infection, jaundice or congenital malformations, or BW <2000 g/>4000 g [8]. It is not possible to further quantify any possible overlap with other conditions from the framework. However, overlap between neonatal respiratory diseases and BW <2000 g should have already been addressed by methods used to estimate BW <2000 g.[5]

5. Severe infection

Evidence source

Sub-Saharan Africa modelled estimate of possible severe bacterial infection (pSBI) for 2012 from a global systematic review by Seale et al.[12] This was selected as the most contextually appropriate estimate given the lack of population-level estimates for Nairobi or similar populations.

Calculations

No initial calculations were required as the estimate was taken directly in its published form.[13]

Overlap adjustment

The Seale et al paper excludes neonates <1500 g and <32 weeks GA. As the authors' cut-off for low BW was <1500 g rather than our <2000 g, some residual overlap is probable. However, methods used to exclude overlap in the BW <2000 g category excluded neonates with other diagnoses, including severe infection (see 'low BW' above). Neonates <1500g BW with severe infection are likely to be missed in our estimates, as they are not counted in either this category or that of BW <2000 g. There is no mention in the paper of excluding neonates with neonatal respiratory diseases, neonatal encephalopathy, neonatal jaundice or congenital malformations. It was not possible to quantify this overlap, or to adjust our outcome with reference to this or other above issues, without detailed insight into the modelling techniques used.

Late onset sepsis

Discussion with the advisory group suggested that most neonates >7 days old with severe infection ('late-onset neonatal sepsis' (LONS)) are likely to be admitted to the paediatric ward rather than the newborn unit, and so, from a health service provision perspective, should not be counted in our framework. For neonatal sepsis the percentage of neonates who present within the first 7 days of life ('early-onset neonatal sepsis' (EONS)) is difficult to define accurately and varies by context.[14,15] Understanding of this for the Nairobi population is limited by the lack of population-level studies of neonatal sepsis in this or similar populations.

The Seale et al systematic review used for estimating severe infection in our estimates [12] does not provide a breakdown of incidence by age at presentation. However, it is likely that the majority of cases are in neonates in the first week of life as many of the included studies focused on this group, and the YICCS criteria were specifically developed for neonates <7 days old. A systematic review by Waters et al of neonatal sepsis aetiology showed 45.4% of 'culture-positive' neonatal sepsis (clinical signs of neonatal infection plus positive urine or blood cultures for bacterial infection) in African studies occurred within the first week of life,[14] but the validity of this as a general ratio is limited by marked variance in culture positivity rates between studies. A large South African study by Cutland et al accounting for 75% of annual births in an urban low- and middle-income population showed early onset neonatal sepsis to account for 60% of culture-proven neonatal sepsis, but 91.2% of neonatal sepsis overall (culture-proven plus clinically diagnosed without positive culture).[16] Overall neonatal sepsis incidence in this study was 3-4% (lower than 6.2% in our estimates). The recent AFRIcan NEonatal Sepsis Trial (AFRINEST) [17,18] observed 41.72% of neonatal pSBI to occur in the first 7 days of life (Simon Cousens, Personal Communication, 2016). Features of study design (enrolment was via community health worker visits within 24 hours of birth; however, a proportion of neonates with severe infection may have died before enrolment) and study population (more than 40% of births were not in health facilities) limit the applicability of these data to our population.

Although the available evidence suggests that those with early-onset neonatal sepsis comprise a large proportion of all neonatal severe infection, the generalisability of existing studies to the Nairobi population and interpretation of results to define the population requiring inpatients services, remains a challenge. Given that we have not been able to exclude those neonates with late-onset neonatal sepsis from our estimate of 62.00 per 1000 live births requiring inpatient care, it is likely that our estimate is an overestimate if only admissions to the newborn unit are to be considered.

Adjusting for late-onset sepsis

If we were to apply the Waters et al estimate of 54.6% of severe infection cases being LONS [14] and exclude these, our estimate of 62.00 per 1000 live births with severe infection would change to 28.15 (*95%CI 18.61-37.68*) per 1000 live births with severe infection in the early neonatal period. The effect of excluding late-onset infection from our overall estimate of newborns requiring inpatient care would be to reduce the estimate to 149.24/1000 live births (*bounds of confidence 125.72-176.15)*. By contrast, if the estimate from Cutland et al [16] of 8.8% of severe infection cases being LONS, our estimates for severe infection requiring inpatient care in the newborn unit would be 56.54 severe infection cases/1000 live births (*95% CI 37.39-75.70*) and the overall estimate would be 177.63/1000 live births *(bounds of confidence 144.49-214.16)* after exclusion of LONS. These alternative scenarios are presented in **Table S3**, described as "high estimate of LONS" and "low estimate of LONS" respectively.

6. Jaundice requiring treatment

Evidence source

The population based-study of 5266 neonates in Nigeria by Olusanya et al [19], was selected as the most appropriate evidence source because it was identified as the largest population-based study of a population similar to the Nairobi population. Overlap adjustment calculations were performed using the sub-Saharan African estimate from a global systematic review of neonatal jaundice.[20]

The Olusanya et al study involved mothers attending primary healthcare clinics for newborn vaccinations in Lagos, Nigeria being asked if there was any history of jaundice in the first week of life necessitating hospital admission for phototherapy (“baby put under light without clothes”) and/or for exchange blood transfusion (“baby’s blood was changed”). Overall vaccine uptake in the study population was estimated to be 75-98%, and these four clinics were known to account for >75% of all vaccination in the city.

To give context, the estimate was compared with high-income studies where treatment criteria are likely to be stricter (although treatment policy has changed over time), acknowledging the large difference in risk factor distribution between high-income settings and our target population. The only large published study that was identified reported 4.0% of live births requiring phototherapy for neonatal jaundice in a large tertiary referral centre in Australia.[21] Experts in the field of neonatal jaundice highlighted the difference in treatment practices between facilities and countries, but advised that between 1% and 4% of neonates in 2 major European countries (Denmark and UK) receive phototherapy (Gorm Griesen, Rasmus Rogvi, Neena Modi, Thomas Williams, personal communications, 2016).

Calculations

No calculations were required as the estimate was taken directly in its published form, being the reported incidence of neonates requiring phototherapy or exchange blood transfusion for neonatal jaundice.[19]

Overlap adjustment

A sensitivity analysis from a systematic review by the UK National Institute of Clinical Excellence (NICE) estimated a minimum of 13.9% of cases of neonatal jaundice in Africa were related to sepsis.[20] This was used to adjust the estimate from the Nigeria community based study and exclude the common comorbidity of severe infection in neonatal jaundice. There were no explicit exclusion criteria in the Olusanya et al study and so overlap may exist with preterm <32 weeks, BW <2000 g, neonatal respiratory conditions, and major congenital malformations. Overlap with BW <2000 g was excluded by methods used to estimate BW <2000 g. This was not possible for other categories.

7. Major congenital malformations

Evidence source

For different congenital malformation groups, data were used from the Modell Global Database of Congenital Disorders (MGDb, developed by Professor Bernadette Modell,[22,23] the International Clearinghouse for Birth Defects Surveillance and Research (ICBDSR),[24] the European Concerted Action on Congenital Anomalies and Twins (EUROCAT),[25] and individual studies from sub-Saharan Africa [26-32] and high-income settings.[33,34] The selection of sources for each congenital malformation group was guided by available data, methodological concerns, and expert advice.

Calculations

There was a notable lack of birth registry or surveillance data for congenital malformations in SSA. In light of this, and as a result of the heterogeneity of estimates from other settings, it was decided to select upper and lower bounds for each congenital malformation group from available estimates. This involved appraisal of evidence sources as for other framework categories, but was also

informed by guidance from experts in the field and current understanding of the risk factors and pathogenesis of individual conditions. Details of conditions included and evidence sources selected for upper and lower bounds for each congenital malformation category are shown in **Table S4** below.

In providing final outcomes it was decided to take a mean of the upper and lower bounds and present this as the final estimate, with the upper and lower bounds as intervals of confidence. In the case of major gastrointestinal malformations, we did not present intervals of confidence, and used our selected estimate as the final estimate, for reasons described in **Table S4**. Means and confidence intervals were summed to provide a final overall outcome for 'major congenital malformations' **(table S4**).

Overlap adjustment

Congenital malformations vary markedly with GA, and are much more prevalent in neonates born preterm. Prevalence ratios were taken from a large collection of birth registries in the United States of America (7,209,768 births)[34] and applied to overall outcomes for congenital malformations to distribute incidence by GA. In adjusting for overlap, incidence of major congenital malformations in neonates <32 weeks GA (65.6% of all incidence) was excluded. None of the sources described above excluded BW <2000 g, neonatal respiratory conditions or jaundice, and so it is likely some overlap remains. Overlap with BW <2000 g was excluded by methods used to estimate BW <2000 g. For other categories it was not possible to quantify overlap.

8. Intrapartum stillbirths

Evidence source

Modelled estimate for Kenya from global systematic review for 2009 were used to estimate overall stillbirths [35] and SSA estimates for 2009 were used to estimate the proportion of stillbirths that were intrapartum [36].

There has been a recently published update of the Lawn et al stillbirth modelled estimates [35,37], using 177% more data points for the SSA region and estimating Kenyan stillbirth incidence for 2015 at 22.5 per 1000 births (previous unadjusted estimate utilised in our study: 21.86 per 1000 live births).[36] Unfortunately neither the paper nor the supplementary data provided estimates of specifically intrapartum stillbirths or national uncertainty ranges, and so given the similarity between the estimates it was decided to use those with greater granularity from the previous round of published estimates.[37]

Calculations

The overall estimate for stillbirths in Kenya was 21.86 per 1000 births;[35] 46.5% of these were estimated by Cousens et al to be intrapartum.[36] By applying this proportion to the overall estimate, an estimate of intrapartum stillbirths was calculated. The same calculation was applied to confidence intervals from the baseline estimate.

Overlap adjustment

As all other sections of the framework focus on conditions occurring in live births and this section explicitly only estimates neonates who die before birth, there should be no overlap. A possible exception to this could be in the case of conditions such as some major congenital malformations or

severe neonatal encephalopathy where neonates die very soon after birth and in some cases might be misclassified as 'stillbirths'. This could result in some double counting/overlap, however it is likely to be minimal and was not possible to quantify. In addition, a proportion of neonates who are stillborn will also have a major congenital malformation; however, this is not necessary to consider as overlap, as the major congenital malformations outcome only estimates live births.

9. Large for gestational age (>4000 g)

Evidence source

Unpublished breakdown of published WHOMCS BW data [1] on 6439 births in 6 hospitals in Nairobi City County, as for 'very preterm' (see above).[2]

Calculations

Individual numbers of births >4000 g BW reported for each of the 6 WHOMCS Nairobi hospitals were summed and divided by the summed total number of births for each facility to give an overall incidence estimate of 28.30 per 1000 live births. 95% confidence intervals were then calculated as 24.50 – 32.60 per 1000 live births.

Overlap adjustment

The same calculation process was performed as described for BW <2000 g: the ratio from Pumwani Maternity Hospital data of those BW >4000 g with no other admission diagnosis apart from "LGA/Macrosomia" to overall births >4000 g BW admitted to the NBU was calculated as 0.838. This ratio was then applied to the overall WHOMCS estimate of births >4000 g BW to calculate a new outcome excluding neonates >4000 g BW with another diagnosis at admission, shown in Table S1.

Data from Pumwani Maternity Hospital suggest that a very high proportion (83.8%) of neonates >4000g will have no other diagnosis, much higher than the percentage of neonates <2000 g with no other diagnosis. It is thought this is likely because neonates >4000 g are less physiologically fragile than those <2000 g,[38] and in this context are primarily being admitted for investigation to prevent longer-term complications (e.g. to exclude maternal diabetes) but not often as a result of acute neonatal illness. This was discussed with the expert advisory group who felt this high proportion of LGA neonates with no other admission diagnosis was likely to lie close to the true incidence for the Nairobi population.

Appendix 2: Supplemental tables

Table S1: Classification of evidence

Evidence Level	Description
I (ideal)	Population-based estimates for Nairobi City County population
II	Large population-based estimates of population similar to the ideal (i.e. Kenya national data or similar sub-Saharan African population)
IIIa	Systematic review of population-based studies providing national estimate for Kenya
IIIb	Systematic review of population-based studies, providing regional estimate for East Africa or sub-Saharan Africa
IV	Individual population-based studies of populations substantially different to the ideal (e.g. population groups from high-income settings)
V (least appropriate)	Facility-based studies

Table S2: Appraisal of estimates

Conditions	Directness	Risk of bias	Imprecision	Inconsistency	Comment from advisory group	GRADE
**<32 weeks preterm**	Nairobi multi-facility study	Facility studies vary on population coverage. Gestational age estimation technique	Minimal	Variance in estimates between hospital sites	**Agree:** estimate likely to lie close to true incidence. Heterogeneity of hospital sites studied in WHOMCS encompassing majority of births in Nairobi major strength of estimate source.	⊕⊕⊕◯
**Birthweight <2000 g**	Nairobi multi-facility study & Nairobi facility study	Facility studies vary on population coverage	Diagnostic overlap difficult to determine	Facility studies report higher incidence compared with population estimates	**High estimate:** Unadjusted estimate likely to be close to true incidence, but the proportion with no co-morbidity likely to be an overestimate.	⊕◯◯◯
**Large for gestational age**	Nairobi multi-facility study & Nairobi facility study	Facility studies vary on population coverage	Minimal	Variance in estimates between hospital sites	**Agree:** estimate likely to lie close to the true incidence.	⊕⊕◯◯
**Neonatal encephalopathy**	SSA modelled estimate	Minimal	Wide confidence intervals	Minimal	**Agree:** neonatal encephalopathy has become less prevalent since the provision of free maternity care; the proposed estimate likely to be close to the true incidence.	⊕⊕⊕◯
**Neonatal Respiratory Diseases**	European population studies	Minimal	Minimal	Minimal	**Low estimate**: estimate for neonates 32-36 weeks GA thought to be lower than that seen in Nairobi, due in part to a lower prevalence of timely antenatal steroid usage in their population. The estimate for those ≥37 weeks was considered appropriate. Variance also noted in other population determinants of neonatal respiratory diseases incidence between European populations and that of Nairobi, for example lower rates of caesarean section in Nairobi.	⊕⊕◯◯
**Severe infection**	SSA modelled estimate	WHO YISSG criteria may overestimate infection	Wide confidence intervals	Minimal	**Low estimate:** those who receive empiric treatment for severe infection might be closer to the upper confidence interval of 8.3% of live births, however acknowledge that not all neonates treated are eventually diagnosed with severe infection.	⊕⊕◯◯
**Jaundice requiring treatment**	SSA community study/SSA modelled estimate	Facility studies vary on population coverage. Small study sizes.	Wide confidence intervals	Variance in estimates between facility studies	**High estimate**: higher than the suggestions of the advisory group (3-4%) and of published high-income estimates. In addition to residual overlap, this could, in part, be due to genetic factors, particularly a lower prevalence of glucose-6-phosphate dehydrogenase deficiency in Kenya compared with Nigeria.	⊕◯◯◯

Conditions	Directness	Risk of bias	Imprecision	Inconsistency	Comment from advisory group	GRADE
**Major congenital malformations**	Kenya modelled estimate/ European population studies/ SSA facility studies	Facility studies vary on population coverage. Inconsistent diagnostics	Unable to estimate confidence intervals for certain outcomes	Heterogeneity in different estimate sources despite apparently consistent genetic predisposition	**Agree:** despite likely overestimation of neural tube defects due to evidence source not taking into account new programme of folic acid supplementation in Kenya, the overall estimate for major congenital malformations is likely to lie close to the true incidence. Other life-threatening congenital malformations are rare, so their exclusion is unlikely to greatly affect this estimate.	⊕⊕◯◯
**Intrapartum stillbirths**	Kenyan modelled estimate	Minimal	Wide confidence intervals	Minimal	**Agree:** likely close to the true incidence, however the possibility that source studies from low-resource settings could have misclassified early neonatal deaths as stillbirths was acknowledged.	⊕⊕⊕◯

Table S3: The effect of alternative admission policies for neonates with late onset neonatal sepsis (LONS) or large for gestational age (LGA) on overall estimates of total admissions for inpatient neonatal care in Nairobi City County

Admission policy scenarios	Admissions (n/1000 live births)	Lower bound	Upper bound	Notes
**Admit LONS and LGA to NBU.**	206.80	168.62	248.77	LGA adjusted estimate 23.71 (95% CI 20.52 - 27.31)
**Admit LONS to NBU, do not admit LGA to NBU.**	183.09	148.10	221.46	This is the final adjusted result presented in 'results' section of the main text.
**Do not admit LONS (low estimate of LONS – Cutland et al [16]) or LGA to NBU.**	177.63	144.49	214.16	Low estimate excludes 8.8% of severe infection resulting in estimate of 56.54 (95% CI 37.39 - 75.70)
**Do not admit LONS (high estimate of LONS – Waters et al [14]) or LGA to NBU.**	149.24	125.72	176.15	High estimate excludes 54.6% of severe infection resulting in estimate of 28.15 (95% CI 18.61 – 37.68)

Definitions: LONS – late onset neonatal sepsis, NBU – newborn unit, LGA – large for gestational age >4000g

All estimates are of total admission numbers and therefore adjusted to exclude overlap between multiple illness episodes if possible (see 'methods' section of main text).

Table S4: Conditions included and estimate sources for individual congenital malformation groupings

Category	Lower Bound (n/1000 live births)	Upper Bound (n/1000 live births)	Mean (n/1000 live births)	Conditions included & estimate sources
Congenital heart defects	1.75	4.72	3.24	It was aimed to only include congenital heart defects likely to result in mortality or severe morbidity without neonatal inpatient care. The MGDb estimate (4.72/1000 live births) was used as an upper bound and the median of two African facility-based studies and estimates of 'critical congenital heart disease' from EUROCAT and a high-income study (1.75/1000 live births) as a lower bound. [28,33,39,40]
Major CNS defects	2.21	2.31	2.26	Encephalocele, spina bifida, anencephaly (collectively referred to as neural tube defects) and hydrocephalus were included. MGDb estimates were used for neural tube defects and these were summed with EUROCAT estimates of hydrocephalus (0.58/1000 live births) as an upper bound.[40] The median of 6 African facility-based studies was used as a lower bound (0.48/1000 live births) [28-32,39], giving an overall incidence estimate of major CNS defects of 2.21-2.31/1000 live births.
Orofacial clefts	0.97	1.31	1.14	Isolated cleft palate, isolated cleft lip and combined cleft lip and palate were included. A multicentre study in Ghana by Agbenorku et al was selected as the upper bound (1.31/1000 live births) [41] and a multicentre study in Uganda by Dreise et al was used for the lower bound (0.97/1000 live births).[26]
Major GI malformations	NA		1.68	Oesophageal atresia/ tracheoesophageal fistula, duodenal atresia, anorectal atresia/stenosis, congenital diaphragmatic hernia and abdominal wall defects (exomphalos & gastroschisis) were included. For this outcome it was decided to use the sum of EUROCAT estimates (1.68/1000 live births),[40] which was the highest estimate of those we compared. This remains slightly lower than actual incidence, in the opinion of expert clinical geneticists and the advisory group, and in light of this it was decided not to have a lower bound for major GI malformations.
**TOTAL**	**6.07**	**10.02**	**8.05**	

Definitions: CNS – Central Nervous System, GI – Gastrointestinal, MGDb – Modell Global Database of Congenital Disorders, EUROCAT – the European Concerted Action on Congenital Anomalies and Twins.

Appendix 3: Estimate specific limitations

1. Very pre-term (<32 weeks)

Although the WHOMCS estimate is from a large birth cohort located specifically in Nairobi City County, it did not capture all births in the county in the time period and there may be some selection bias due both to some neonates not being born in facilities and also the specific facilities included/excluded. Despite this, it is a large, multi-centre study, and was selected as the best available estimate for Nairobi City County.

Method of GA assessment is a significant issue in most studies of GA in low-income settings.[4] In WHOMCS, GA was assessed using *'best obstetric estimate'*, the description of this in the study protocol is as follows: *"accurate GA estimation may depend on the date of onset of the last normal period, pelvic examination, obstetric examination and obstetric ultrasound examination or another parameter used. These elements are evaluated at delivery and an overall estimation is made. If there is no obstetrical data available, use the recorded estimate of GA by neonatal physical examination."* This variance in methods of GA assessment is a potential source of error. Using the first day of last menstrual period to estimate GA is known to overestimate prematurity and be vulnerable to reporting inaccuracy, whilst neonatal physical examination has been shown to underestimate the GA of very premature infants by as much as 2 weeks compared with ultrasound.[4,42] Ultrasound is generally considered to be the most precise GA dating method, however unfortunately is rarely available in SSA.[4] This may go some way to explaining the notable variance in incidence of prematurity between hospital sites, with one large public hospital reporting an incidence of neonates born <32 weeks GA of 16.1/1000 live births, compared with a smaller, private hospital reporting an incidence of 6.8/1000 live births.

2. Birthweight <2000 g

See 'very pre-term' section above for discussion of WHOMCS strengths/limitations as an evidence source. BW estimation is less technically difficult than GA estimation and therefore typically more robustly estimated.[2,43] Pumwani Maternity Hospital data are from a single tertiary referral centre and are only of neonates admitted to the NBU. Therefore, it is important to consider how generalisable these data are to the population of Nairobi as a whole. Additionally, these data may be limited by information bias caused by incomplete documentation of data in medical records by clinicians. The estimate (from Pumwani data) that 45.8% of all births BW <2000 g have no diagnosis (40.04% of births <1800g) other than BW <2000 g was felt to be overly high by the advisory group. If this is the case, our estimate with overlap excluded may be an overestimate.

3. Neonatal encephalopathy

The authors report one limitation of their estimates was the high variability in case definition for severity of neonatal encephalopathy from data sources [5]. The exact methods used to overcome this are unclear from the publication; however, it seems that severity was allocated from each source using Sarnat grading as a guide.[5,44]

4. Neonatal respiratory diseases

Although each study used for this section was of a high methodological standard with large sample size, the marked differences in populations between those studied and that of Nairobi County raise important concerns regarding the transferability of estimates. This results in a poor score for 'directness' in the GRADE framework. Notable differences in risk factors include caesarean section prevalence, maternal age at delivery, GA at birth, BW, and timely antenatal steroid use in preterm

births. The Altman et al study in Sweden used to estimate RDS only reported incidence in neonates ≥30 weeks GA [6,7]. RDS becomes increasingly common with increasing prematurity and is most common in those <30 weeks gestational age. As these neonates are not counted, our composite outcome for RDS/TTN in those <32 weeks GA is likely to be an underestimate.

5. Severe infection

The Seale et al paper acknowledges concerns that the use of the YICCS criteria may result in the inclusion of some non-severe infections. However, a benefit of the use of YICCS criteria is that its broad nature likely resulted in the inclusion of neonates with severe non-bacterial infections (i.e. viral or fungal), which may require inpatient neonatal care.

Neonates <1500g BW with severe infection are likely to be missed in our estimates, as they are not counted in either this category or that of BW <2000 g.

6. Jaundice requiring treatment

There are several important limitations to the Olusanya et al study.[19] Firstly, any neonates who died from jaundice, or another condition, before BCG immunisation, were missed. Secondly, the outcome was a retrospective review of which neonates had phototherapy, not of serum bilirubin or other clinical parameter. Thus, the proportion of neonates receiving phototherapy who did not require it ('overtreatment'), or those who should have received it and did not ('undertreatment') was not possible to ascertain. Thirdly, jaundice occurring in the late neonatal period (>7 days of life) was not captured. However, the advisory group advised that, as for neonates with severe infection, those with late-onset neonatal jaundice are typically admitted to paediatric wards not the NBU, and so should not be counted in our framework. As we were not able to estimate late neonatal jaundice we were not able to present alternative scenarios displaying the impact of this admission policy.

Our estimated outcome of 5.77-6.99% requiring treatment is higher than the suggestions of the advisory group (3-4%) and of published high-income estimates[20] and expert opinion (see Appendix 1 section 'jaundice requiring treatment'), thus, decreasing confidence in our estimate. In addition to the above methodological concerns and residual overlap described in **Appendix 1**, these inconsistencies could, in part, be due to genetic factors, particularly a lower prevalence of glucose-6-phosphate dehydrogenase (G6PD) deficiency (one of the main risk factors for neonatal jaundice) in Kenya compared with Nigeria.[21]

7. Major congenital malformations

The advisory group provided guidance with regards to which congenital malformations are typically admitted for inpatient neonatal care in the Kenyan setting. This guidance was incorporated when selecting which congenital malformations to include in the framework outcome. This resulted in some conditions being excluded from the framework, which would normally be diagnosed during inpatient neonatal admission in a high-income setting. For example Hirschprung's disease was excluded from the framework, as in Kenya this typically presents in children older than 28 days of age and so is not managed through inpatient neonatal services.[37] There are numerous rare congenital malformations, some of which will likely result in physiological decompensation in the neonatal period requiring hospitalisation. Due to their rarity, and the likelihood that many of these might be counted as another diagnosis due to limited diagnostic facilities, efforts are not made to include these here, although, they are explicitly acknowledged in the 'miscellaneous conditions' section of our framework.

The Kenyan Demographic and Health Survey (DHS) 2014 suggests 74.7% of mothers took iron or folic acid supplementation in their last pregnancy.[45] MGDb estimates do not take into account the impact of a nationwide folic acid food supplementation programme started in 2012. Resultantly, our estimate for neural tube defects may be too high.

8. Intrapartum stillbirths

Our estimate from Lawn et al is based on the international standard definition of third trimester stillbirths of ≥28 weeks gestational age and ≥1000g BW [35]. Thus our estimate does not include earlier gestation foetal deaths, some of which would be considered viable pregnancies in high-income settings.

9. Large for gestational age

See 'very pre-term' section above for discussion of WHOMCS strengths/limitations as an evidence source. See 'birthweight <2000 g' section above for discussion of robustness of BW estimation and limitations of data from Pumwani Maternity Hospital.

References

1. Morisaki N, Togoobaatar G, Vogel JP, et al. Risk factors for spontaneous and provider-initiated preterm delivery in high and low Human Development Index countries : a secondary analysis of the World Health Organization Multicountry Survey on Maternal and Newborn Health. *British Journal of Obstetrics and Gynaecology* 2014; **121**: 101-9.
2. Souza JP, Gulmezoglu AM, Vogel J, et al. Moving beyond essential interventions for reduction of maternal mortality (the WHO Multicountry Survey on Maternal and Newborn Health): a cross-sectional study. *Lancet* 2013; **381**(9879): 1747-55.
3. Blencowe H, Cousens S, Oestergaard MZ, et al. National, regional, and worldwide estimates of preterm birth rates in the year 2010 with time trends since 1990 for selected countries: A systematic analysis and implications. *The Lancet* 2012; **379**: 2162-72.
4. Marchant T, Willey B, Katz J, et al. Neonatal Mortality Risk Associated with Preterm Birth in East Africa, Adjusted by Weight for Gestational Age: Individual Participant Level Meta-Analysis. *PLoS medicine* 2012; **9**.
5. Lee AC, Kozuki N, Blencowe H, et al. Intrapartum-related neonatal encephalopathy incidence and impairment at regional and global levels for 2010 with trends from 1990. *Pediatric research* 2013; **74 Suppl 1**: 50-72.
6. Altman M, Vanpée M, Cnattingius S, Norman M. Neonatal morbidity in moderately preterm infants: A swedish national population-based study. *Journal of Pediatrics* 2011; **158**.
7. Altman M, Vanpée M, Cnattingius S, Norman M. Risk factors for acute respiratory morbidity in moderately preterm infants. *Paediatric and Perinatal Epidemiology* 2013; **27**: 172-81.
8. Balchin I, Whittaker JC, Lamont RF, Steer PJ. Maternal and fetal characteristics associated with meconium-stained amniotic fluid. *Obstetrics and gynecology* 2011; **117**: 828-35.
9. Fanaroff AA. Meconium aspiration syndrome: historical aspects. *J Perinatol* 2008; **28 Suppl 3**: S3-7.
10. Yurdakok M. Meconium aspiration syndrome: do we know? *Turk J Pediatr* 2011; **53**(2): 121-9.
11. Newcombe RG. Two-sided confidence intervals for the single proportion: comparison of seven methods. *Stat Med* 1998; **17**(8): 857-72.
12. Seale AC, Blencowe H, Manu AA, et al. Estimates of possible severe bacterial infection in neonates in sub-Saharan Africa , south Asia , and Latin America for 2012 : a systematic review and meta-analysis. *The Lancet Infectious Diseases* 2014; **3099**: 1-11.

13. Seale AC, Blencowe H, Zaidi A, et al. Neonatal severe bacterial infection impairment estimates in South Asia, sub-Saharan Africa, and Latin America for 2010. *Pediatric research* 2013; **74 Suppl 1**: 73-85.
14. Waters D, Jawad I, Ahmad A, et al. Aetiology of community-acquired neonatal sepsis in low and middle income countries. *Journal of global health* 2011; **1**: 154-70.
15. Huynh BT, Padget M, Garin B, et al. Burden of bacterial resistance among neonatal infections in low income countries: how convincing is the epidemiological evidence? *BMC Infect Dis* 2015; **15**: 127.
16. Cutland CL, Madhi SA, Zell ER, et al. Chlorhexidine maternal-vaginal and neonate body wipes in sepsis and vertical transmission of pathogenic bacteria in South Africa: a randomised, controlled trial. *The Lancet* 2009; **374**: 1909-16.
17. AFRIcan NEonatal Sepsis Trial Group. Simplified regimens for management of neonates and young infants with severe infection when hospital admission is not possible: study protocol for a randomized, open-label equivalence trial. *Pediatr Infect Dis J* 2013; **32 Suppl 1**: S26-32.
18. Esamai F, Ayede AI, Adejuyigbe EA, Tshefu A, Wammanda RD, African Neonatal Sepsis Trial g. Simplified antibiotic regimens for neonatal sepsis--AFRINEST - Authors' reply. *Lancet* 2015; **386**(10001): 1339.
19. Olusanya BO, Akande AA, Emokpae A, Olowe SA. Infants with severe neonatal jaundice in Lagos, Nigeria: incidence, correlates and hearing screening outcomes. *Trop Med Int Health* 2009; **14**(3): 301-10.
20. Aride C, Barron J, Benjamin Y, et al. Neonatal jaundice. 2010; London; 2010. p. 1-495.
21. Guaran RL, Drew JH, Watkins AM. Jaundice: clinical practice in 88,000 liveborn infants. *Aust N Z J Obstet Gynaecol* 1992; **32**(3): 186-92.
22. Modell B, Berry R, Boyle Ca, et al. Global regional and national causes of child mortality. *The Lancet* 2012; **380**: 1556.
23. Modell B, Darlison M, Moorthie S, Blencowe H, Petrou M, Lawn J. Epidemiological Methods in Community Genetics and the Modell Global Database of Congenital Disorders (MGDb). 2016. http://discovery.ucl.ac.uk/1532179/.
24. International Centre on Birth Defects. International Clearinghouse for Birth Defects and Surveillance Annual report 2012. 2012; 2012.
25. EUROCAT. EUROCAT: European Surveillance of Congenital Anomalies. 2015.
26. Dreise M, Galiwango G, Hodges A. Incidence of Cleft Lip and Palate in Uganda. *The Cleft Palate-Craniofacial Journal* 2011; **48**: 156-60.
27. Agbenorku P, Yore M, Danso Ka, Turpin C. Incidence of Orofacial Clefts in Kumasi, Ghana. *ISRN Plastic Surgery* 2013; **2013**: 1-6.
28. Delport SD, Christianson AL, Berg HJSVD, Wolmarans L, Gericke GS. Congenital anomalies in black South African liveborn neonates at an urban academic hospital. *South African Medical Journal* 1995; **85**: 11-5.
29. Ekanem TB, Bassey IE, Mesembe OE, Eluwa MA, Ekong MB. Incidence of congenital malformation in 2 major hospitals in Rivers state of Nigeria from 1990 to 2003. *Eastern Mediterranean Health Journal* 2011; **17**: 701-5.
30. Muga R. Congenital Malformations among Newborns in Kenya. *African Journal of Food Agricultture, Nutrition and Development* 2009; **9**: 814-29.
31. Venter PA, Christianson AL, Hutamo CM, Makhura MP, Gericke GS. Congenital anomalies in rural black South African neonates - a silent epidemic? *South African Medical Journal* 1995; **85**: 15-20.
32. Msamati B, Igbigbi P, Chisi J. The incidence of cleft lip, cleft palate, hydrocephalus and spina bifida and Queen Elizabeth Central Hospital, Blantyre, Malawi. *Central African Medical Journal* 2000; **46**: 292-6.
33. Peterson C, Ailes E, Riehle-Colarusso T, et al. Late Detection of Critical Congenital Heart Disease Among US Infants. *JAMA Pediatrics* 2014; **168**: 361.

34. Honein Ma, Kirby RS, Meyer RE, et al. The association between major birth defects and preterm birth. *Maternal and Child Health Journal* 2009; **13**: 164-75.
35. Lawn JE, Blencowe H, Pattinson R, et al. Stillbirths: Where? When? Why? How to make the data count? *The Lancet* 2011; **377**: 1448-63.
36. Cousens S, Blencowe H, Stanton C, et al. National , regional , and worldwide estimates of stillbirth rates in 2009 with trends since 1995 : a systematic analysis. *The Lancet* 2011; **377**: 1319-30.
37. Project TP. Hirschsprung ' s Disease in Africa in the 21 st Century. 2011; Toronto; 2011. p. 1-27.
38. Blencowe H, Cousens S, Jassir FB, et al. National, regional, and worldwide estimates of stillbirth rates in 2015 with trends from 2000: A systematic analysis. *The Lancet Global Health* 2016; **4**: e98-108.
39. Ndibazza J, Lule S, Nampijja M, et al. A Description of Congenital Anomalies Among Infants in Entebbe , Uganda. *Birth Defects Research* 2011; **861**: 857-61.
40. EUROCAT. EUROCAT Prevalence Data Tables. 2012.
41. Agbenorku P. Orofacial Clefts: A Worldwide Review of the Problem. *ISRN Plastic Surgery* 2013; **2013**: 1-7.
42. Lynch C, Zhang J. The research implications of the selection of a gestational age estimation method. *Paediatric and perinatal epidemiology* 2007; **21**: 86-96.
43. World Health Organization. WHO recommendations on interventions to improve preterm birth outcomes. 2015; Geneva; 2015.
44. Sarnat HB, Sarnat MS. Neonatal encephalopathy following fetal distress. A clinical and electroencephalographic study. *Arch Neurol* 1976; **33**(10): 696-705.
45. Kenya National Bureau of Statistics. 2014 Kenya Demographic and Health Survey (2014 KDHS). Nairobi, Kenya: Kenya National Bureau of Statistics, 2015.
